# Lipid accumulation and biosynthesis genes response of the oleaginous *Chlorella pyrenoidosa* under three nutrition stressors

**DOI:** 10.1186/1754-6834-7-17

**Published:** 2014-01-30

**Authors:** Jianhua Fan, Yanbin Cui, Minxi Wan, Weiliang Wang, Yuanguang Li

**Affiliations:** 1 State Key Laboratory of Bioreactor Engineering, East China University of Science and Technology, 130 Meilong Road, 301 Mail box, Shanghai 200237, PR China

**Keywords:** Nitrogen, Phosphorus and iron deficiency, Lipid accumulation, Physiological response, Gene expression, *Chlorella*

## Abstract

**Background:**

Microalgae can accumulate considerable amounts of lipids under different nutrient-deficient conditions, making them as one of the most promising sustainable sources for biofuel production. These inducible processes provide a powerful experimental basis for fully understanding the mechanisms of physiological acclimation, lipid hyperaccumulation and gene expression in algae. In this study, three nutrient-deficiency strategies, viz nitrogen-, phosphorus- and iron-deficiency were applied to trigger the lipid hyperaccumulation in an oleaginous *Chlorella pyrenoidosa*. Regular patterns of growth characteristics, lipid accumulation, physiological parameters, as well as the expression patterns of lipid biosynthesis-related genes were fully analyzed and compared.

**Results:**

Our results showed that all the nutrient stress conditions could enhance the lipid content considerably compared with the control. The total lipid and neutral lipid contents exhibit the most marked increment under nitrogen deficiency, achieving 50.32% and 34.29% of dry cell weight at the end of cultivation, respectively. Both photosynthesis indicators and reactive oxygen species parameters reveal that physiological stress turned up when exposed to nutrient depletions. Time-course transcript patterns of lipid biosynthesis-related genes showed that diverse expression dynamics probably contributes to the different lipidic phenotypes under stress conditions. By analyzing the correlation between lipid content and gene expression level, we pinpoint several genes viz. *rbsL*, *me g6562*, *accA*, *accD*, *dgat g2354*, *dgat g3280* and *dgat g7063*, which encode corresponding enzymes or subunits of malic enzyme, ACCase and diacylglycerol acyltransferase in the *de novo* TAG biosynthesis pathway, are highly related to lipid accumulation and might be exploited as target genes for genetic modification.

**Conclusion:**

This study provided us not only a comprehensive picture of adaptive mechanisms from physiological perspective, but also a number of targeted genes that can be used for a systematic metabolic engineering. Besides, our results also represented the feasibility of lipid production through trophic transition cultivation modes, throwing light on a two-stage microalgal lipid production strategy with which heterotrophy stage provides sufficient robust seed and nitrogen-starvation photoautotrophy stage enhances the overall lipid productivity.

## Background

Rapid exploitation of fossil fuels in modern society has been widely recognized as highly unsustainable. The limited storage and the contribution of fossil fuels combustion to air pollution are two main concerns that current energy consumption status bring about [1]. Biofuel from photosynthetic microorganisms was regarded as an ideal strategy to produce renewable energy. Microalgae are among the most photosynthetically efficient species on earth; the lipid amount produced per acre by algae greatly exceeds that of agricultural oleaginous crops. Moreover, the capacity of sequestering greenhouse gas also entitles them to be one of the most promising sustainable sources for biofuel production [2,3].

However, many challenges have to be resolved before the industrial-scale application of microalgal biofuel becomes cost-effective, one of which is the lack of microalgal strains with both high lipid content and fast growth rate. System metabolic engineering is widely recognized as a promising solution to address this issue because it can manipulate metabolic flux to generate more precursors for triacylglycerol (TAG) accumulation [4,5]. There have been several reports on the use of systems metabolic engineering in developing microbial hosts for the production of biofuels [6,7], while to our best knowledge, few successful reports are available on microalgae. Though bioinformatics provides a predictive tool for analyzing and comparing similar biochemical routes in already sequenced microalgal genomes [8], little experimental validation of putative enzyme activities has so far been reported. Therefore, more studies need to be systematically performed to improve our understanding of these organisms with respect to genetics and physiology, especially on the molecular mechanisms of fatty acid and TAG metabolism. In this study, three enzymes concerning the central carbon metabolism pathway were detected, viz. l,5-ribulose bisphosphate carboxylase/oxygenase (RuBisCO), malic enzyme (ME) and phosphoenolpyruvate carboxylase (PEPC), which mainly regulate the fixation of CO_2_, the supply of nicotinamide adenine dinucleotide phosphate (NADPH) and the competition for the substrate pyruvate, respectively. In addition, a series of genes encoding two enzymes of committing steps in the lipid biosynthesis pathway were examined as well: acetyl-CoA carboxylase (ACCase) and acyl-CoA, and diacylglycerol acyltransferase (DGAT), which catalyze the first and the final committed step in TAG biosynthesis, respectively. Numerous studies reported an enhanced lipid accumulation by genetically modified genes encoding these enzymes in many other species [9,10], whereas little success has been achieved using similar methods in microalgae.

It has been well-documented that under nutrient stress, microalgae cells would accumulate cellular lipid level to 50% to 70% of the biomass, with TAGs accounting for the dominant proportion [11]. Normally, trapped light energy and fixed inorganic carbon would primarily be stored as carbohydrates; the metabolic flux would channel to TAGs when such stresses occur, but the underlying lipid biosynthesis molecular mechanism of this methodology is poorly understood. With respect to metabolic engineering, obtaining information about differentially expressed genes under these conditions is conducive to selecting key targets that can be addressed in the engineering process for increased TAG production. Potential target genes for strain improvement can be identified by comparing the expression levels of genes between strains of different phenotypes under different environmental conditions [12]. In fact, most key enzymes in the lipid biosynthesis pathway possess many an isoenzyme or subunit that is encoded by different genes with expression patterns that vary dramatically from each other under stimuli [13]. The understanding of how microalgae respond to nutrient stress at molecular level is largely limited to several model species, such as *Chlamydomonas reinhardtii*[14,15], *Thalassiosira pseudonana*[16] and *Dunaliella salina*[17], which were not under direct consideration for the production of biomass as a biofuel feedstock. There are scant reports available on algae species with a background of industrial exploitation.

Green algae *Chlorella* spp. has been one of the earliest commercially developed algal species. It provides a high proportion of protein and contains an impressive amount of vitamins and minerals, pigments, fatty acids, and growth factor. Current research has also demonstrated its potent viability for biofuel production [11,18,19]. A robust algal species *Chlorella pyrenoidsa* (*C. pyrenoidosa*) has been well-studied by our group and multiple cultivation strategies were developed for efficient algal biomass and lipid production [20,21]. Currently, this alga is exploited as an industrial strain for both *Chlorella* powder and algal fuels production in the scale-up system. Moreover, the genomic sequencing of this algal strain has been completed (http://www.ncbi.nlm.nih.gov/bioproject/PRJNA171991), which facilitated the study on lipid biosynthesis at molecular level. In this study, three nutrient-deficient strategies (nitrogen, phosphorus and iron deficiency) were applied as the stimuli to trigger the TAG hyperaccumulation in an industrial strain of *C. pyrenoidosa*. The objectives of this study were to pinpoint transcriptional profiles of key genes related to the biosynthesis pathways of TAG as well as to examine the regular patterns of the growth, photosynthesis activity and reactive oxygen status in response to different nutrient stress in oleaginous algae.

## Results and discussion

### Growth characteristics and lipid accumulation of *C. pyrenoidosa* subject to different nutrient deprivation conditions

**Figure 1:**
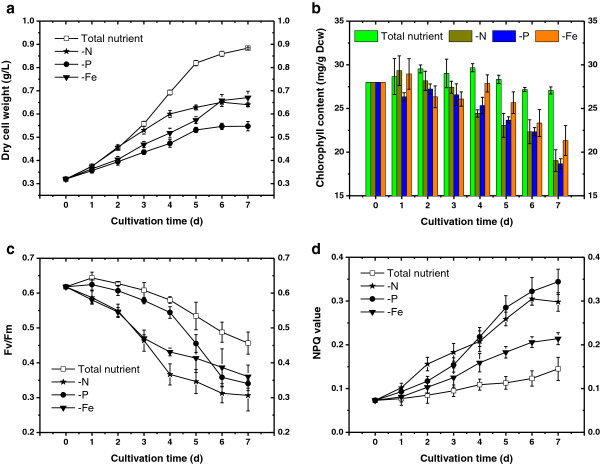
**Growth and photosynthetic activity of *Chlorella pyrenoidosa *under different stress conditions. (a)** Growth, **(b)** chlorophyll content, **(c)** variable-to-maximum fluorescence ratio (Fv/Fm) and **(d)** NPQ value. N, nitrogen; P, phosphorus; Fe, iron.

**Figure 2:**
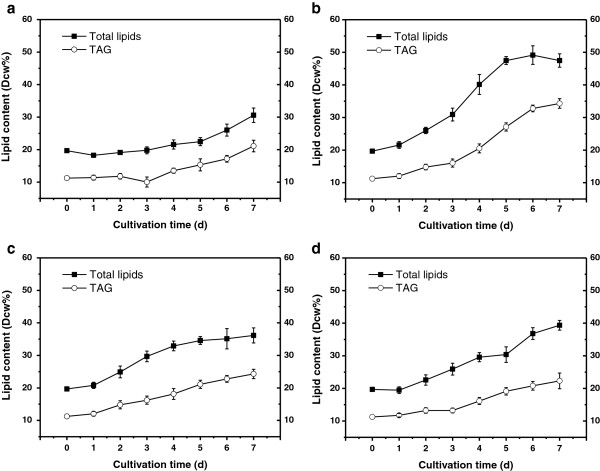
**The lipid content of *Chlorella pyrenoidosa*.** Lipid content under **(a)** total nutrient cultivation, **(b)** nitrogen-deficient cultivation, **(c)** phosphorus-deficient cultivation and **(d)** iron-deficient cultivation. TAG, triacylglycerol.

Heterotrophic algal cells were collected and inoculated into modified F/2 medium for total nutrient cultivation and into nutrient-deficient medium (N-free, P-free and Fe-free) for starvation cultivation with an initial biomass density of 0.32 g/L. As indicated in Figure 1a, the algal cell density under total nutrient condition exhibited fast and continuous growth, obtaining a cell density of 0.88 g/L at the end point. The initial total lipids content and neutral lipids content of algal cells was 19.69% and 11.23%, respectively; the lipids content of total nutrient condition remained constantly low during the initial 5 days with a noticeable increase in the last two days (Figure 2a).

Consistent with many other studies conducted under nutrient stress, the cell growth was arrested and the lipid content exhibited an early rise compared with the control. The phosphorus (P)-deficient cultivation had the most severe influence on the growth of *C. pyrenoidosa*, obtaining a final cell density at only 0.55 g/L, whereas the lipid content only experienced a modest increase (Figure 2c). Phosphorus is a constituent element of ATP and is essential for photophosphorylation, which has significant relevance to the cell growth and metabolism of microalgae. Of particular relevance, photosynthesis requires large amounts of proteins (mainly RuBisCO) which are synthesized by phosphorus-rich ribosomes [22]. Accordingly, phosphate depletion had a severe impact on various aspects of microalgal metabolism and led to a drastic drop in biomass density compared with the control. Nitrogen is the most abundant element of intracellular components as well as the primary constituent of most macromolecules. Hence, it is reasonable to observe no significant differences between total nutrient and nitrogen (N)-free medium in the initial growth rate until day 3; the nitrogen reservoir could sustain the cell growth for a few days (Figure 1a). After the fourth day, the cell growth was severely hindered; meanwhile, the total lipid content and neutral lipids content increased sharply from 30.91% and 16.03% to 49.14% and 32.78%, through day 3 to day 6, respectively (Figure 2b). Numerous reports have demonstrated N-starvation enhances lipids, mainly TAG accumulation in many microalgae species [11,23]. In this study, both the total lipid content and neutral lipid content exhibited the most marked increase under N-deprivation, with 50.32% and 34.29% of dry cell weight at the end of cultivation, respectively. For iron (Fe)-deprivation, the influence on cell growth and lipid accumulation seems to be mild; the final biomass was higher than that of P-depletion and the final lipid content was lower than that of N-depletion (Figure 1 and Figure 2). Interestingly, in many other reports, high iron level was conducive to lipid accumulation [24,25], whereas in our study the lipid content witnessed an impressive increase when exposed to iron deficiency, compared with total nutrient cultivation; the possible reason might be the heterotrophic algal seed, with iron storage sufficient for the cell to sustain cell metabolism but insufficient for cell division. It has been commonly noticed that when a nutrient element is insufficient for the protein synthesis required by growth, excess carbon from photosynthesis would be channeled into such storage molecules as TAGs [26]. CO_2_ fixation decreases due to direct effects on photosynthetic structures such as chlorophyll, light-harvesting complex and RuBisCO. These possible reasons can be further verified by the results concerning photosynthetic activity.

Lipid productivity is of particular importance in large-scale microalgal lipid production processes because it takes into account both lipid content and biomass production rate. The results suggest that the nitrogen starvation strategy can result in the most favorable lipid productivity (Tables 1 and 2). It can be determined that the highest cumulative lipid productivity was obtained at day 5 (47.05 mg/L/d), hence the fifth day is recommended for biomass harvest for the highest lipid production by this cultivation mode. Though all the nutrient deficient cultivations repressed the growth of algal biomass, the overall productivity caused by nitrogen deficiency was not offset by biomass loss. In fact, compared with most previous studies of algal cellular lipid accumulation by means of nutrient starvation, the nitrogen-deficient cultivation mode in our study exhibited superiority in lipid productivity. The trophic transition from heterotrophy to autotrophy might be a critical reason that contributes to lipid overproduction, as the heterotrophic algal seeds exhibited superiority in both biomass growth and lipid accumulation in the subsequent photoautotrophic cultivation [21]. Similarly, Fan found that the transition from heterotrophy to photo-induction culture can stimulate a dramatic increase of lipid in three *Chlorella* species [20]; Zheng also discovered that heterotrophic *Chlorella sorokiniana* (*C. sorokiniana*) performs much better for lipid production in large-scale open systems than its phototrophic counterpart [27]. Our results hereby provide a hint for a two-stage microalgae lipid-production mode, which with the heterotrophy stage provides sufficient robust seed, and the nitrogen-starvation autotrophy stage enhances the overall lipid productivity.

**Table 1:** **Cumulative lipid productivities (mg/L/d) of *Chlorella pyrenoidosa *** **under different culture conditions**

**Cultivation mode**	**Day 1**	**Day 2**	**Day 3**	**Day 4**	**Day 5**	**Day 6**	**Day 7**
Full medium	5.41	11.80	15.80	21.61	24.21	26.77	29.59
Nitrogen-deficient	17.70	28.05	33.64	44.45	47.05	42.79	34.42
Phosphorus-deficient	11.04	17.61	22.12	23.17	24.15	21.69	19.27
Iron-deficient	7.79	14.14	19.42	22.58	22.23	29.88	28.67

**Table 2:** **Cumulative TAG productivities (mg/L/d) of *Chlorella pyrenoidosa *** **under different culture conditions**

**Cultivation mode**	**Day 1**	**Day 2**	**Day 3**	**Day 4**	**Day 5**	**Day 6**	**Day 7**
Full medium	6.81	8.76	6.67	14.45	17.92	18.62	21.50
Nitrogen-deficient	9.09	15.92	16.36	21.81	26.88	29.56	26.24
Phosphorus-deficient	6.93	11.23	11.59	12.45	15.25	14.88	13.88
Iron-deficient	6.76	8.74	8.66	11.92	14.74	16.81	16.21

### Chlorophyll content and chlorophyll a fluorescence transient

The chloroplast is the fundamental unit for most photosynthetic plants and algae, hence the content of chlorophyll and the vitality of the photosynthetic machine are critical physiological indicators through which we can inspect the algal cell adaptation when exposed to nutrient-deficiency. As was indicated in Figure 1b, the chlorophyll content of algal cells in full medium cultivation fluctuated around 28 mg/g in first 4 days, followed by a slight decrease in the stationary phase, whereas nutrient-deficient cultivations resulted in early decrease in chlorophyll content. Both nitrogen deficiency and phosphorus deficiency caused a sharp decline in chlorophyll content, reaching about 18 mg/g at day 7. The degradation caused by iron deficiency was relatively less significant at the end of culture where the chlorophyll content was 21.8 mg/g. Nitrogen and phosphorus deprivation led to the degradation of the highly abundant light-harvesting proteins so that they could be used as sources for protein synthesis [28]. As the consumption of nitrate and phosphate is much larger than that of iron, algal cells have to degrade more synthetic machines to keep cell growth in the N-deficient and P-deficient conditions. A marked decrease in chlorophyll content was also witnessed in *Chlamydomonas* when exposed to a nitrogen starvation condition [13], which could be explained by the nutrient recycling from the degradation of anabolic structural components when exogenous nutrient is depleted. Our results also showed that all nutrient-deficient treatments would lead chlorophyll degradation. Most of the anabolic machinery, including ribosomes and chloroplast, become superfluous when nutrient deficient conditions cannot sustain cell growth; those structural components would be targeted for degradation and nutrient recycling. The recycled excess carbon likely provides substrates and energy for the synthesis of storage compounds such as neutral lipids [29].

The time course of maximum photochemical efficiency of photosystem II and non-photochemical quenching were measured during the cultivation and revealed significant differences between control and stressed cultures (Figure 1c and d). The variable-to-maximum fluorescence ratio (Fv/Fm) ratio was measured to evaluate changes in the photosynthesis efficiency of algal cell under different treatments. In the control group, the Fv/Fm ratio increased slightly on the first day then decreased gradually to 0.46 on day 7. The exposure of algal cells to nutrient starvation led to more dramatic decline than in the control. N-deficient and Fe-deficient cells exhibited an early declining pattern with the terminal value equal to 0.30 and 0.36, respectively. However, the downgrade caused by P deficiency was less significant until the fifth day; Fv/Fm eventually dropped to 0.34 on the seventh day.

**Figure 3:**
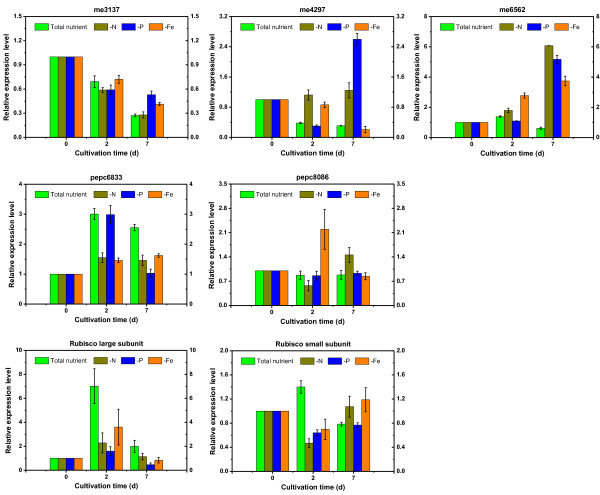
**Expression level of l,5-ribulose bisphosphate carboxylase/oxygenase (RuBisCO), malic enzyme (ME) and phosphoenolpyruvate carboxylase (PEPC) genes under nutritional deprivation conditions.** N, nitrogen; P, phosphorus; Fe, iron.

The decline in the Fv/Fm ratio value under all nutrient deficiencies indicates that the photosynthesis efficiency of algal cells was undermined due to lack of constructional matters for maintaining the photosynthesis machinery. It has also been demonstrated that nutrient stress would lead to a decline in the Fv/Fm ratio; when the limiting nutrient was added into the medium it would increase markedly [30]. Nitrogen depletion was detrimental to the photosynthesis efficiency, mainly due to the large demand for this element in cell growth. Additionally, drastic downregulation in the abundance of light-harvesting complexes might be another reason that contributes to reduction of photosynthesis activity [31]. In another part of this study, we examined the transcript of genes encoding the subunits of RuBisCO; the results indicated that the relevant transcript level of both large and small subunits exhibited an obvious drop under nutrient-deficient conditions compared to that of the total nutrient culture (Figure 3). A similar trend can also be determined in phosphorus and iron depletion treatments, with a less notable range. These results as a whole suggest that the depletion of nutritional elements in the medium resulted in a significant decline in chlorophyll content and photosynthetic activity.

### Reactive oxygen species content and antioxidant response

**Figure 4:**
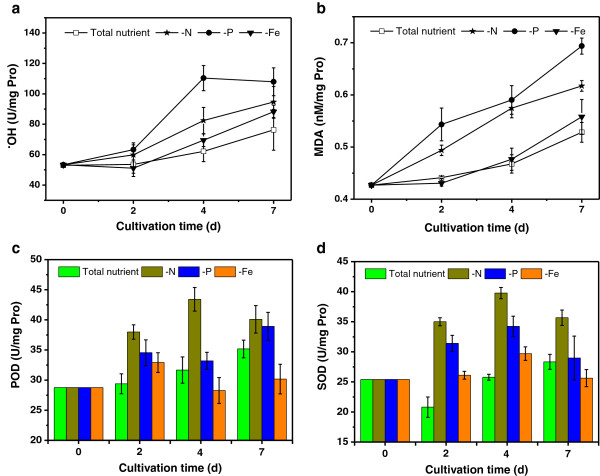
**Hydroxide radical (OH) and malondialdehyde (MDA) content, and the peroxidase (POD) activity and superoxide dismutase (SOD) activity of Chlorella pyrenoidosa under different nutrient limitations. (a)**⋅OH, **(b)** MDA, **(c)** POD, **(d)** SOD. N, nitrogen; P, phosphorus; Fe, iron.

In this part, we investigated physiological parameters to confirm the toxicological effects of the three nutrient stressors, including the level of reactive oxygen species (ROS) and the activities of antioxidant enzymes like superoxide dismutase (SOD) and peroxidase (POD). Plants and algae can activate several defense systems for scavenging ROS, but under unfavorable conditions the generation rate of ROS out-paces the scavenging rate, thus the excess ROS causes massive damage in the cell. In the present study, we compared the content of hydroxyl radicals and membrane lipid peroxidation as well as activities of ROS eliminating enzymes (Figure 4) to further understand the ROS level and antioxidant capacity triggered by nutrient stress.

The hydroxyl radical (OH) is a highly toxic form of reactive oxygen intermediate and malondialdehyde (MDA) is an indicator of membrane lipid peroxidation; the content of both items at the starting point were 53 U/mgPro and 0.43 nM/mgPro, respectively. In total nutrient condition, both items stayed at a low level during the initial stage and increased slightly after the fourth day. The ⋅OH content (Figure 4) in the P-deficient condition increased sharply after cultivation for 2 days, whereas in N deficiency and Fe deficiency it exhibited an approximately linear increase after the second day: the terminal ⋅OH content of those treatments were 108 U/mgPro (P-deficient), 97 U/mgPro (N-deficient) and 88 U/mgPro (Fe-deficient), respectively. MDA content showed a continuous rise in all nutritional treatments, exhibiting a corresponding relevance with that of ⋅OH content at the end of culture, with 0.68 nM/mgPro in P-deficient, 0.63 nM/mgPro in N-deficient and 0.56 nM/mgPro in Fe-deficient cells. It can be figured out that the cell growth curve has an inverse trend with that of ⋅OH and MDA content, which indicates that the growth retardation might be caused by high ROS toxicity. Researchers have indicated that excessive ROS may cause irreversible oxidative damage to macromolecules and activate signaling pathways, ultimately leading to cell death [32]. Counterintuitively, the enzyme activity of both SOD and POD under Fe depletion was at the same level or even lower than that of total nutrient conditions at the late phase of cultivation. Here we postulate that the content of antioxidant enzymes is relevant to both the reactive oxygen stress and the building block that constitutes those enzymes. It has been well-studied that Fe is a crucial component in ferroporphyrin of POD heme binding domain [33], as well as key metal ions in the Fe-SOD SOD isoforms [34].

Recent evidence indicates that ROS are not only associated with cell death, but also play important roles in signaling and cellular adaptation to stress [35]. Autophagy is required for the degradation of damaged or toxic materials that are generated as a result of ROS accumulation, which allows eukaryotic cells to recycle intracellular components under nutrient limitation; degradation and recycling of macromolecules via autophagy provides a source of building blocks [36]. The chloroplast is considered the most powerful source of ROS in plants [37], so it is reasonable that in our study the photosynthetic activity was inversely correlated with ROS level (Figures 1c and 4) to some extent. Furthermore, as a response mechanism to unfavorable surroundings, ROS in the plant cell can also trigger a series of genes coping with environmental stress, mainly by the means of controlling signal transduction pathways. Suzuki demonstrated that absence of nutrients is a primary signal leading to autophagy activation in eukaryotes, but this stress signal is tightly associated with the production and accumulation of ROS, which might trigger the expression of genes to cope with the stress [38]. In many studies, the hyperaccumulation of lipid is regarded as a mechanism to pull through stress conditions [8,14,39]. Therefore, our data indicated that the ROS may play dual roles in leading to both cell death and acclimation through regulation of the expression of specific genes [40,41].

### Expression of lipid biosynthesis genes in *C. pyrenoidosa* subject to nitrogen, phosphorus and iron deprivation

In order to target the genes related to TAG accumulation and learn more about the transcriptional changes in response to nutrient limitation in microalgae, we made an initial effort to search differentially expressed genes using the suppression subtractive hybridization approach under trophic transition: the results indicated that the transition from heterotrophy to photo-induction culture can stimulate lipid accumulation in *Chlorella* coupled with a series differentially expressed genes in the lipid metabolic pathway [42].

**Figure 5:**
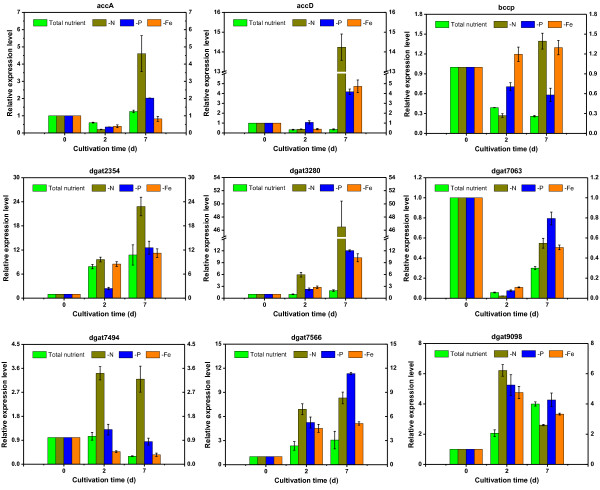
**Expression level of acetyl-CoA carboxylase (ACCase) and diacylglycerol acyltransferase (DGAT) genes under nutritional deprivation conditions.** N, nitrogen; P, phosphorus; Fe, iron; bccp, biotin carboxyl carrier protein.

In the present study, we made a further comparison of the transcript levels of *C. pyrenoidosa* in nutrient stress conditions with those of non-induced, nutrient-replete culture: samples at the starting point (day 0), exponential growth phase (day 2) and stationary phase (day 7) were collected for quantitative exploration. A series of genes in the central carbon metabolism pathway and TAG biosynthesis pathway were monitored, providing a comprehensive insight into how the cell is adapting to a specific condition as well as studying molecular changes that promote TAG hyperaccumulation. The expression patterns of genes that encode isoenzymes or subunits of key enzymes were detected and the results illustrated that some regular patterns can be determined in response to nutrient stress conditions (Figure 3 and 5).

As shown in Figure 3, the transcript level of RuBisCO small subunit under total nutrient condition was relatively constant and the variation between nutrient-deficient treatments and the control was minor, indicating that the *rbcS* might be constitutive expression in algal cells. In contrast, the expression abundance of *rbcL* in the log phase (day 2) was much higher than than in the beginning and the late phase and the expression level of *rbcL* in the total nutrient condition was two to five times higher than that of nutrient-deficient conditions. It has also been indicated by Wan that the expression level of *rbcL* in *C. sorokiniana* was vulnerable to external nutrition conditions [43]. Such a trend also coincided with the growth characteristics, it is logical that the fast algal cell-growth in the total nutrient condition needs more fixed carbon for cell construction, which asks for more RuBisCO to sequester the CO_2_ in the air. The chlorophyll content and photosynthetic activity of algal cells under nutrient-deficient treatments and the total nutrient condition can also verify this cause.

Malic enzyme (ME) was considered to be a major supplier of NADPH which is critical to intracellular fatty acid content [44]. In the present study we investigated three homologous genes for malic enzyme in *C. pyrenoidosa*. The expression level of *me* g6562 enhanced dramatically at the stationary phase under all nutrient deficient conditions, while the abundance of *me* g6562 mRNA in the control almost remained at the same level. For *me* g4297, the transcript abundance increased under phosphorus and nitrogen-starvation cultivation, enhanced 8.43 and 4.04 times at the stationary phase, respectively. In contrast, for *me* g3137, the difference between control and treatments was comparatively minor; the expression of this gene for both the treatments and control exhibited a downregulation over the period. Many studies indicated that ME plays a key role in lipid biosynthesis: in *Mortierella alpine* the ME activity was in correlation with lipid accumulation [45], and the overexpression of genes encoding ME in *M. circinelloides* could enhance lipid content 2.5-fold [46]. In our study, we verified the relation between gene expression and lipid content and provided an insight into the potential targets.

We also detected two genes encoding phosphoenolpyruvate carboxylase (PEPC) in *C. pyrenoidsa*, *pepc* g6833 and *pepc* g8086. The expression levels of *pepc* g8086 at nutrient-deficient conditions were almost equal to that of the total nutrient condition, except for Fe deficiency at the second day, with double the expression level of the control. For *pepc* g6833, mRNA abundance in nutrient-deficient conditions were around half of the control except for P deficiency on the second day, which exhibited the same level as that of the total nutrient condition. PEPC is a key enzyme in the competing pathway of lipid accumulation; blocking off genes encoding this enzyme has enhanced TAG biosynthesis in many species [47,48]. Recently, it was also found that in *C. reinhardtii*, oil content significantly increased after N deficiency, which was accompanied by a drastic decrease in the transcript level of *pepc2*[49]. In our study, the expression level of *pepc* g6833 showed a similar variation, though the range was comparatively minor.

Acetyl-CoA carboxylase (ACCase) catalyzes the first rate-limiting step in the fatty-acid biosynthetic pathway through the formation of malonyl-CoA from acetyl-CoA [50]. Compared with control, N deficiency led to a dramatic upregulation for three subunits in the stationary phase, at 3.5-fold for *accA*, 38.9-fold for *accD* and 6.7-fold for *bccp*; P deficiency and Fe deficiency could also trigger the increase of their expression level, though less impressively. It can also be determined that *accA* and *accD* exhibited a drastic increase in the late stage, whereas for *bccp* the variation was not obvious. Specifically, it can be figured out from Figure 2b that total lipid exhibits a slight decline whereas the expression levels of *accA* and *accD* are still high on day 6. It seems that a secondary limiting-step occurs after fatty acid formation, which prevents the conversion to lipid in algal cells. The reduction of total lipid might also due to the degradation of cell membrane caused by lipid peroxidation which can be validated by malondialdehyde (MDA) content (Figure 4b).

With regard to diacylglycerol acyltransferase, we identified six putative genes in the *C. pyrenoidosa* genome dataset; for most of them (g2354, g3280, g7566, g9098) the expression level during the stationary phase was higher than that of the log phase, which demonstrated the positive role of increased DGAT activity in lipid hyperaccumulation. In terms of the effect upon gene transcript, N deficiency is still the most potent approach for inducing *dgat* expression in the lipid accumulation stage; a 24.5-fold and 8.5-fold upregulation for *dgat* g3280 and *dgat* g7494 were detected in expression level on day 7, respectively. Three genes (g3280, g7063 and g7566) responded sensitively to all nutrient-deficient conditions during the late stage, which suggested their close relevance to lipid biosynthesis. It is worth noting that in *C. pyrenoidosa*, five genes encoding DGAT2 isoform (except g7494) and their expression patterns are similar to those demonstrated by Msanne, who found the expression of DGAT2 in *C. reinhardtii* cells also increased dramatically when subjected to nitrogen starvation [13]. Another study showed that the abundance of DGTT1 mRNA (belonging to type-2 DGAT) increased in nitrogen starvation conditions and exhibits correlation with TAG accumulation in *C. reinhardtii*[31]. These findings were consistent with observations in our study; type-2 DGAT was proven to have a crucial role in lipid accumulation under a nutrient-depletion culture. In particular, we also pinpointed several genes that respond to nutrient stress sensitively: in order to interrogate the relevance between their expressions and lipid accumulation, we further conducted correlation analysis.

### Correlation between gene expression abundance and lipid content

It is ubiquitous that many key enzymes in the lipid biosynthesis pathway possess different isoenzymes. In relation to diacylglycerol acyltransferases (DGAT) for example, there are six genes coding for this enzyme in *Chlamydomonas*, and the expression level of each gene varies dramatically under the nitrogen deprivation condition [13]. Therefore, selecting the exact genes that encode critical isoforms in the lipid biosynthesis pathway is important for genetic engineering, whereas in most previous studies the expression patterns of different key enzymes are confined to a single gene, which might fail to identify the authentic targets for prospective transgenic modification.

In this study, we examined the expression patterns of a series of enzymes responsible for the TAG metabolic pathway using quantitative real-time PCR and carried out Spearman correlation analysis (using SPSS 19.0) to determine the quantitative relationship between the expression level of these genes and lipid content under different nutrient-deficient conditions, thus providing an overall perspective on the underlying mechanism of the TAG hyperaccumulation response to nutrient stress. The relationship between their expression abundance and the lipid synthesis was analyzed according to the results that are presented in Table 3. Several genes that may play a primary role in lipid accumulation were selected as promising candidates for further metabolic engineering.

**Table 3:** Correlation coefficients between gene expression and lipid content

**Gene id**	**Total lipid**	**TAG**	**Gene id**	**Total lipid**	**TAG**
*rbcL*	-0.833*	-0.905**	*accD*	0.786*	0.810*
*rbcS*	0.190	0.143	*bccp*	0.429	0.429
*me* g3137	-0.833*	-0.786*	*dgat* g2354	0.905**	0.857**
*me* g4297	0.190	0.262	*dgat* g3280	0.810*	0.786*
*me* g6562	0.619	0.595	*dgat* g7063	0.762*	0.857**
*pepc* g6833	-0.429	-0.452	*dgat* g7494	-0.048	-0.048
*pepc* g8086	0.048	0.190	*dgat* g7566	0.619	0.690
*accA*	0.690	0.762*	*dgat* g9098	-0.214	-0.190

*P-value <0.05, ** *P*-value <0.01.

RuBisCO plays a key role in the carbon concentration mechanism and is one of the most important enzymes to influence the carbon flux input. The expression level of *rbcL* was in inverse proportion to the lipid content under nutrient-deficient conditions, which indicates this gene manipulates a committed step in photobiosynthesis and further affects cell growth. It can also be determined from Table 3 that several genes, viz. *me g6562*, *accA*, *accD*, *dgat g2354*, *dgat g3280* and *dgat g7063* showed significant correlation with lipid accumulation, these genes encode corresponding isoenzymes of ME, ACCase and DGAT in the *de novo* TAG biosynthesis pathway. These genes are likely to exert great influence on lipid biosynthesis and were selected as modification candidates.

The previous parts of the present study showed that nutrient stress, including nitrogen, phosphorus and iron depletion, could induce considerable lipid accumulation as well as drastic physiological stress in *C. pyrenoidosa*. In this part, we elucidated the transcription alteration of many genes that closely relate to lipid biosynthesis. These results as a whole suggest that some metabolic pathways redirected to the lipid accumulation in *C. pyrenoidosa* were probably triggered when physiological stress was induced by nutrient starvation. The investigation into whether different isoforms confer significantly different phenotypes to algal cells provides an informative cue for future efforts in genetic modification.

## Conclusion

In this study we made an investigation into the cell growth and lipid accumulation along with the transition from heterotrophy to three nutrient-deficiency cultivations, and compared the regular patterns of physiological parameters and transcriptional data between different types of nutrient starvation. The results indicate that physiological stress, including photosynthesis depression and ROS toxicity, were observed after the onset of three nutrient-deficient conditions, especially for nitrogen and phosphorus depletions. Furthermore, the correlation between lipid content and gene expression patterns reveals that several critical genes encoding RuBisCO, ME, ACCase and DGAT are highly related to lipid accumulation, which provides a clue for prospective metabolic engineering. The study not only provides us with a comprehensive picture of adaptive mechanisms from a physiological perspective, but also with a number of targeted genes that can be used for systematic metabolic engineering. Furthermore, our results also represent the feasibility of lipid production through trophic transition cultivation-modes, among which heterotrophy-nitrogen deprivation is suggested as the best way for the two-stage lipid production method.

## Materials and methods

### Algae strain, seed preparation and culture conditions

*C. pyrenoidosa* (FACHB 9) was purchased from the Institute of Hydrobiology, Chinese Academy of Sciences (Wuhan, China) and purified aseptically for further study. Seed cultured heterotrophically in a 500-mL Erlenmeyer flask containing 200 mL Endo medium [51] was placed on a reciprocating shaker (150 rpm) and maintained at 30°C for 3 days. After centrifugation the cell suspension was inoculated into air-lift column photobioreactors (60 cm high and 5 cm diameter) containing 1 L modified F/2 medium (NaNO_3_ 74.5 mg, NaH_2_PO_4_ · 2H_2_O 17.6 mg, 1 mL trace metal solution, 1 mL vitamin solution, and 1,000 mL distilled water). In nutrient-stress cultures, the algae cells were resuspended in N-free, P-free and Fe-free medium, respectively. The culture condition was maintained at 25°C under continuous fluorescent lamps, with light intensity approximately 100 μmol/m^2^/s with one side-illumination. Photobioreactors were aerated with filtered ambient air at a flow rate of 0.5 vvm; CO_2_ from air was the only available source of carbon for the algal cell.

### Measurement of biomass concentration and lipid content

The biomass density was measured by dry cell weight. Whatman GF/C glass fiber micro-filters (Sigma, China) were dried for 2 h at 105°C, cooled in a desiccator and weighed: 10 to 20 mL of the sample was filtered and was dried and cooled before weighing again. Biomass concentration was obtained by the difference between the two weights.

Total lipids were extracted following the protocol reported by Bligh and Dyer [52] with some modifications. The cells (75 to 100 mL) were collected by centrifuging at 5,000 rpm for 10 minutes and freeze-dried for 24 h. Weighed samples were extracted with a solvent mixture of chloroform and methanol (2:1, v/v) for 40 minutes. After centrifuging at 5,000 rpm for 10 minutes, the extracts (supernatant) were collected into a pre-weighed dry glass tube and evaporated by nitrogen flow. The procedure was repeated three times when the organic solvent had no color, which indicated that the total lipids were extracted, and the residue and glass tube were further dried in an oven at 105°C for 12 h and cooled until the weight was constant. Lipid content was calculated by the difference between the two weights and was expressed as % of dry cell weight. For the measurement of neutral lipid content, we referred to the method described by Chen [53], with sample concentration, Nile red concentration and incubation time optimized. Specifically, 0.5 mL of the algal sample was washed three times and diluted by double-distilled water. The final algal sample for neutral lipid content measurement was 0.10 (optical density (OD)_680_). Then, 157 μL final algal sample was transferred into a black 96-well plate containing 40 μL dimethyl sulfoxide (DMSO) aqueous solution and 3 μL of 50 μL/mL Nile red (Sigma, China). The black 96-well plate was rotated at 120 rpm and stained at 30°C in the dark for 10 minutes. After samples were stained, neutral lipids were measured using ELISA (GENios Pro, Tecan, Germany) with an excitation wavelength of 485 nm and an emission wavelength of 590 nm. The neutral lipid standard triolein (Sigma, China), which ranged from 2.0 to 100.0 μg/mL, was used to generate a standard curve.

The images were acquired with a laser scanning confocal microscope (Olympus Fluo ViewTM FV1000, Japan), using an 100 × oil immersion lens, and analyzed with the Fluoview10 (v2.0) software. Laser excitation was at an emission wavelength of 485 nm and Nile red fluorescence was detected between 558 and 600 nm using band-pass filtering. In the presence of nonpolar lipids, Nile red emits a yellow-gold fluorescence (λmax = 580 nm) [54].

### Physiological parameter analysis

The contents of total chlorophyll were determined using a modified method described by Sükran [55]. The centrifuged pellets were resuspended in 96% ethanol and vortexed to extract pigments. Cellular debris was pelleted by centrifugation and chlorophyll-a and -b levels were determined spectrophotometrically, in the supernatant, by measuring optical absorbance at 645 and 663 nm.

The maximum photochemical efficiency of photosystem II of algal samples was detected by measuring variable-to-maximum fluorescence ratio (Fv/Fm) according to the operating procedures on the Dual-PAM-100 (Walz, Effeltrich, Germany). The details of the operating parameters are as follows: irradiance of the measuring light 12 μmol/m^2^/s, saturation pulse 4,000 μmol/m^2^/s, actinic light 126 μmol/m^2^/s. The samples were kept in the dark for 15 minutes before measurement. The original fluorescence (F0) was determined under the irradiance of measuring light. A saturation pulse was applied to obtain maximum fluorescence (Fm) in the dark-adapted samples. All experiments were conducted in triplicate. The effective PS II quantum yield was calculated as follows:

$$\mathrm{Fv}/\mathrm{Fm}=\left(\mathrm{Fm}-F0\right)/\mathrm{Fm};\mathrm{NPQ}=\left(\mathrm{Fm}-F’m\right)/F’m$$

Fresh algal pellet was ground in a mortar with a pestle under lipid nitrogen. The homogenate was centrifuged at 13,000 *g* for 15 minutes. The supernatant was recovered for determinations of MDA, SOD, and POD activity with assay kits (Nanjing Jiancheng Bioengineering Institute).

### RNA isolation and real-time quantitative RT-PCR

Samples were collected at day 0, day 2 and day 7. RNA was isolated according to the Trizol RNA extraction procedure (Invitrogen, USA). The quantity and quality of the isolated total RNA was assessed by electrophoresis on denatured agarose gel and by Nano-Drop 3.0 (Coleman Technologies Inc., USA). The RNA solutions were aliquoted and stored at -80°C if not immediately used. The first-strand cDNA synthesis was carried out using the gDNA Remover Reverse Transcription Kit (Toyobo, Japan) according to the manufacturer’s instruction.

Using gene sequences retrieved from NCBI genome sequencing databases, primers of different homologs of key enzymes in the TAG synthesis pathway were designed and used for expression-level quantitative detection. An additional file shows primers for each target gene in more detail (see Additional file 1: Table S1). Real-time quantitative PCR was performed using SYBR Green Realtime PCR Master Mix (Toyobo, Japan) on the C1000 Thermal cycler Real-Time PCR Detection System (Bio-Rad, USA). The actin gene from *C. pyrenoidosa* was used as an internal control to normalize differences between the loading amounts of the template. All PCR reactions were carried out within a final volume of 20 μL containing 1 μL of cDNA template, 1.5 μL of 10 mM deoxyribonucleotide triphosphates, 1 μL of each primer (10 mM), and 10 μL of SYBR Mix. The PCR parameters were set as follows: 28 cycles of 94°C for 30 s, appropriate annealing temperatures for 30 s, and 72°C for 1 minute, with an additional initial 5-minute denaturation at 94°C and a 5-minute final extension at 72°C. All procedures were performed according to the manufacturers’ instructions. Additional file 1: Table S1 summarizes the primer sequences used in this study. The 2^-ΔΔCT^ method was used to analyze the fold change in gene expression relative to control.

### Statistical analysis

All exposure experiments were repeated three times independently, and data were recorded as the mean with SD. For gene expression experiments, quantitative real-time PCR analysis was performed using the BioRAD software. For each gene, the fold change expressed as the mean ± SD (% control) was calculated using the (standard curve) approximation corrected for primer efficiency and normalized to the house-keeping gene, actin, expression values. Statistical analyses were performed using Spearman correlation analysis (SPSS19.0). For all of the data analysis, a *P*-value <0.05 was considered statistically significant.

## Abbreviations

accA: acetyl-CoA carboxylase α carboxyltransferase; ACCase: acetyl-CoA carboxylase; accD: acetyl-CoA carboxylase β carboxyltransferase; bccp/BCCP: biotin carboxyl carrier protein; dgat/DGAT: diacylglycerol acyltransferase; DGTT: type-2 diacylglycerol acyltransferase; ELISA: enzyme-linked immunosorbent assay; Fv/Fm: variable-to-maximum fluorescence ratio; MDA: malondialdehyde; me/ME: malic enzyme; NADPH: nicotinamide adenine dinucleotide phosphate; OH: hydroxyl radical; pepc/PEPC: phosphoenolpyruvate carboxylase; POD: peroxidase; rbsL: ribulose bisphosphate carboxylase oxygenase large subunit; ROS: reactive oxygen species; RuBisCO: l,5-ribulose bisphosphate carboxylase/oxygenase; SOD: superoxide dismutase; TAG: triacylglycerol.

## Competing interests

The authors declare that they have no competing interests.

## Authors’ contributions

JF, YC, YL, WW designed research; YC, JF, WW performed research; YC, JF analyzed data; YC, JF, YL, WW, MW wrote the paper. All authors read and approved the final manuscript.

## Supplementary Material

Additional file 1: Table S1 Quantitative PCR primers for each target gene. Click here for file
