# Supplementary material for: Lipid accumulation and biosynthesis genes response of the oleaginous Chlorella pyrenoidosa under three nutrition stressors
Source: Biotechnol Biofuels. 2014 Jan 30;7:17. doi: 10.1186/1754-6834-7-17 (PMC3916312; doi:10.1186/1754-6834-7-17)
Supplement: Additional file 1: Table S1 — Quantitative PCR primers for each target gene. [file 1754-6834-7-17-S1.docx]

**Table S1 Q-PCR Primers for each target gene**

| Name of enzyme | Gene ID | Primer sequence | product length |
| --- | --- | --- | --- |
| *accA* | g9519 | F: CGCAATACCAAGGAGAACAT | 202bp |
|  |  | R: ACATCTCACGCAGGTTGAC |  |
| *accD* | chloroplast gene | F: TAGTTTGTGCTTCGGGTGG | 227bp |
|  |  | R: CAATAAGGGCTTTCGGTTCA |  |
| *bccp* | g2736 | F: GGCACCAATGTTATGACAGG | 271bp |
|  |  | R: AAGGTGCTGGTGGAGAATG |  |
| *rbcL* | chloroplast gene | F: CTTTCCAAGGTCCTCCTCAC | 208bp |
|  |  | R: TCTCTCCAACGCATAAATGG |  |
| *rbcS* |  | F: GGACTACATCGTGAACAACG | 131bp |
|  |  | R: TCCAGTAGCGGTTGTCGTAG |  |
| *dgat* | g2354 | F: TTTGGCGAGAATGAGCAGTA | 236bp |
|  |  | R: ACCTTTGAAGGCGGGCAG |  |
| *dgat* | g3280 | F: GGCACAAAGAGTTCACCGT | 145bp |
|  |  | R: ACAAACTTGAGGTGGGTG |  |
| *dgat* | g7063 | F: CGGTGTTTGCTTTCGGACAGA | 147bp |
|  |  | R: GGCACAGCTCTTGAATGTACT |  |
| *dgat* | g7494 | F: GCTCGCTGGGCCTGATGCTGTT | 186bp |
|  |  | R: GCGGATGAGCGGGAAGTAGA |  |
| *dgat* | g7566 | F: GGGTGTCCCAACCTCAGAT | 217bp |
|  |  | R: AGCAGCATAGTCAGCAGTGG |  |
| *dgat* | g9098 | F: CGCTTCCTGAGGTTTAGCAT | 194bp |
|  |  | R: CGGCACCACATCGGCAGAGTA |  |
| *me* | g3137 | F: CCCTCTCGTTCCCCTTTTATT | 158bp |
|  |  | R: AAATGCTGACGCAAGTGTGA |  |
| *me* | g4297 | F: GGAGACAATGAAGGCAGCAG | 218bp |
|  |  | R: GGAGGTGTAGGTTGGGAAGAA |  |
| *me* | g6562 | F: CGTTGTTGTACGCCTTGTTG | 227bp |
|  |  | R: GTGAGGACATGCTGATTGAGG |  |
| *pepc* | g6833 | F: AAGGAGTGGGACGAGGATAAG | 238bp |
|  |  | R: GGTGTGGGACATTGAGATGAT |  |
| *pepc* | g8086 | F: GACTATCCCCTTCAGCCACTC | 185bp |
|  |  | R: AAACAGCTCCTCAGCCATCTT |  |
| *actin* |  | F: GCTCAACTCCTCCACGCT | 187bp |
|  |  | R: GTCCTTGCGGATGTCCAC |  |
